# Cancer survival among children of Turkish descent in Germany 1980–2005: a registry-based analysis

**DOI:** 10.1186/1471-2407-8-355

**Published:** 2008-11-28

**Authors:** Claudia Spix, Jacob Spallek, Peter Kaatsch, Oliver Razum, Hajo Zeeb

**Affiliations:** 1 German Childhood Cancer Registry (GCCR), Institute for Medical Biostatistics, Epidemiology and Informatics (IMBEI), Obere Zahlbacher Straße 69, 55131 Mainz, Germany; 2 Department of Epidemiology & International Public Health, School of Public Health, Bielefeld University. P.O. Box 10 01 31, 33501 Bielefeld, Germany; 3 Institute for Medical Biostatistics, Epidemiology and Informatics (IMBEI), Obere Zahlbacher Straße 69, 55131 Mainz, Germany

## Abstract

**Background:**

Little is known about the effect of migrant status on childhood cancer survival. We studied cancer survival among children of Turkish descent in the German Cancer Childhood Registry, one of the largest childhood cancer registries worldwide.

**Methods:**

We identified children of Turkish descent among cancer cases using a name-based approach. We compared 5-year survival probabilities of Turkish and other children in three time periods of diagnosis (1980–87, 1988–95, 1996–2005) using the Kaplan-Meier method and log-rank tests.

**Results:**

The 5-year survival probability for all cancers among 1774 cases of Turkish descent (4.76% of all 37.259 cases) was 76.9% compared to 77.6% in the comparison group (all other cases; p = 0.15). We found no age- or sex-specific survival differences (p-values between p = 0.18 and p = 0.90). For the period 1980–87, the 5-year survival probability among Turkish children with lymphoid leukaemia was significantly lower (62% versus 75.8%; p < 0.0001), this remains unexplained. For more recently diagnosed leukaemias, we saw no survival differences for Turkish and non-Turkish children.

**Conclusion:**

Our results suggest that nowadays Turkish migrant status has no bearing on the outcome of childhood cancer therapies in Germany. The inclusion of currently more than 95% of all childhood cancer cases in standardised treatment protocols is likely to contribute to this finding.

## Background

Mortality from childhood cancer has been declining for almost all cancers in most industrialised countries. 5 year-survival probabilities have been improving between 30% and 50% for childhood cancers evaluated by European registries when comparing the period 1978–1982 to the period 1993–1997 [1]; survival probabilities have improved further in more recent years (see for example data from ). This is due to increasingly effective and highly standardised therapeutic regimes. Country specific analyses in the Automated Childhood Cancer Information System (ACCIS) database show marked survival probability variations especially between childhood cancer in Eastern and Western-European populations (overall survival 1988–97 East: 62%, West: 75%) [1,2], indicating an influence of socioeconomic conditions at the national level.

Socioeconomic status has long been known to influence cancer survival [3]. Migrants and their families may be particularly affected by these effects because of difficulties to obtain and maintain appropriate access to care [4]. However, little is known about childhood cancer incidence and survival among migrants. Hemminki & Li [5] found increased cancer risks for migrant children of different origins and for different diagnoses compared to Swedish children. Data from Great Britain indicate that in 1991–92 children with South Asian ethnic background had an increased cancer incidence when compared to non South Asians [6]. For Germany no incidence comparisons exist, but evaluations of proportional cancer incidence ratios [7] showed little variation between children of Turkish descent as compared to other children.

Few data exist on survival probabilities of migrants. Overall survival seems not to differ between ethnic groups in Great Britain [8,9], with a possible exception of acute lymphoid leukaemia with lower survival probability among UK children of South Asian ethnic background. In the US, there is conflicting evidence concerning ethnic differences in survival probability after childhood acute lymphoid leukaemia [10-12]. For acute myeloid leukaemia the survival probability of US black and Hispanic children is lower than for white children [13].

Apart from ethnic German immigrants from the former USSR, Turkish people are the largest migrant group in Germany, comprising about 1.7 million people in 2007 according to nationality [14], and about 2.4 million when all persons with Turkish migration history are taken into account, including naturalised immigrants and their children [15]. We aimed to study whether a migration background affects survival from childhood cancer in Germany. Therefore, we undertook a registry-based analysis of cancer survival of children with Turkish descent in Germany, using data from the German Childhood Cancer Registry (GCCR) for the period 1980–2005.

## Methods

The GCCR is one of the largest cancer registries worldwide dedicated to children. Annually, about 1800 new cases under 15 years of age are recorded, and the completeness of registration exceeds 95% for all cancer types except central nervous system (CNS) tumours, where completeness is somewhat lower as expected by inter-registry comparisons. No information on ethnicity, and consequently on completeness by ethnicity is available. The GCCR started registering cases in 1980. The current degree of completeness was reached around 1987. Registration with full identification is possible with parental consent, and almost all cases are registered with names. In addition, parents' names are available for about 50% of all cases. A vital status follow-up is conducted routinely using information from various sources (clinical studies, hospitals, communities, families). Close cooperation exists between paediatric oncologists and the registry. As almost all children with cancer in Germany enter into clinical trials, this cooperation substantially adds to the high completeness and quality of the registry [16]. Diagnoses are coded according to the International Classification of Diseases for Oncology ICD-O-3 and classified by the International Classification of Childhood Cancer ICCC-3 [17].

The methods for the retrospective identification of cases of Turkish descent in the registry have been described elsewhere in detail [7]. In short, the routinely collected registry data does not allow systematically determining patients' ethnic or geographical origins. We therefore used a validated name-based algorithm [18,19] to identify Turkish cases by name. The respective study base were all registered cancer cases during the period 1980–2005 for whom the full name was available (n = 37,259, 95% of all registered cases). The automated name – algorithm procedure was augmented by a manual search done by a research assistant with proficiency in Turkish language and names to check the validity of the automated procedure [20]. This procedure has been shown to identify cases of Turkish descent as indicated by their name (referred to as Turkish cases) with a high sensitivity (>97.5%) and specificity (>99%) compared to a complete manually checked gold standard. In order to maintain the high degree of confidentiality required, all data base work was performed at the premises and under surveillance of the GCCR.

No information was available regarding migrant generation, but it is likely that especially the more recently diagnosed cases contain a large proportion of descendants of migrants from Turkey who came to Germany in the 1960s.

We assessed the average duration and completeness of follow-up and specifically the percentage lost to follow-up from the routinely collected data for children of Turkish and non-Turkish descent.

We calculated total and diagnosis-specific 5-year survival probabilities for different strata of age, sex and period of diagnosis (1980–87, 1988–95, 1996–2005) for Turkish and non-Turkish (largely German) childhood cancer patients, using the Kaplan-Meier method. The endpoint was death from any cause. Follow-up ended at the end of 2006 or at loss to follow-up. The statistical significance of differences in survival probability was assessed by the log-rank test for the total survival curve. Analyses were restricted to diagnoses with at least five deaths among registered cases of Turkish descent. We used the SAS statistical software package (SAS, Cary, N.C.) for the analyses. Approval was obtained from the Ethics Committee of the Medical Chamber Westfalen-Lippe.

## Results

Among 37,259 cases in the registry for whom names were available, we identified 1774 cases (4.76%) with Turkish descent as indicated by their name. Characteristics such as sex and age distribution were similar among the two comparison groups, but loss to follow-up was somewhat higher among the cases of Turkish descent (Table 1). Slight differences in the age distribution reflect the different age distributions of the diagnoses, where Turkish cases are slightly more frequent in some diagnosis subgroups. Amongst those diagnosed until 2000 and not reported dead at the end of 2006, the percentage of cases with Turkish descent whose follow-up exceeded 5 years was slightly smaller (95.8%) than among the non-Turkish registry population (98.5%). Overall, loss to follow-up of these cases before the end of 2006 was higher among children with Turkish ethnicity (12.6% against 5.6% in the comparison population). This is mostly due to former patients moving back to Turkey at a later age. The fraction of Turkish cases was close to the average 4.8% for most diagnoses. Notable exceptions were acute myeloid leukaemias, chronic myeloproliferative diseases, myelodysplastic syndrome and other myeloproliferative disease, lymphomas and reticuloendothelial neoplasms, and malignant gonadal germ cell tumours with slightly higher proportions of Turkish cases. We found rather lower proportions of Turkish cases for specified intracranial and intraspinal neoplasms, retinoblastoma, renal tumours, other specified soft tissue sarcomas, germ cell tumours, trophoblastic tumours and neoplasms of gonads and other malignant epithelial neoplasms and malignant melanomas. The range of proportions is 3.4% to 9.0% (see Additional file 1). For a more detailed discussion of these differences see [7].

**Table 1:** Baseline characteristics and quality of follow up in the two groups 1980–2005

	Turkish descent N (%)	Not Turkish N (%)
Number of cases	1774	35,485

male sex	1042 (58.7)	19,822 (55.9)

Age at diagnosis		
<1 year	167 (9.4)	3555 (10.0)
1–<5 years	682 (38.4)	12,914 (36.4)
5–<10 years	503 (28.4)	9516 (26.8)
10–<15 years	422 (23.8)	9500 (26.8)

Year of diagnosis		
1980–87	369 (20.8)	7576 (21.4)
1988–95	477 (26.9)	11,366 (32.0)
1996–2005	928 (52.3)	16,543 (46.6)

Median length of follow-up of patients still alive (range) in years	7.1 (0–28)	8.4 (0–28)

Deaths by 31.12.2006	456 (25.7)	8737 (24.6)

**Follow-up by period of diagnosis**		

1980–1995		
Alive, at least 10 years f/u	507 (92.0)	12,613 (96.7)
Alive, lost to f/u	93 (16.9)	910 (7.0)

1980–2000		
Alive, at least 5 years f/u	879 (95.8)	19,549 (98.5)
Alive, lost to f/u	116 (12.6)	1104 (5.6)

1996–2000		
Alive, at least 5 years f/u	357 (97.3)	6725 (98.8)
Alive, lost to f/u	23 (6.3)	194 (2.9)

N = number, f/u = follow-up

Overall, 25.7% of the cases of Turkish descent (n = 456) had died until 2006 compared to 24.6% of cases (n = 8737) with non-Turkish background.

For cases diagnosed between 1980 and 2005, the 5-year survival probability was 76.9% for cases of Turkish descent and 77.6% for the comparison group (log-rank test: p = 0.15). We found no sex or age-specific differences between the two groups in the overall survival probabilities over the full time period 1980–2005 (Table 2).

**Table 2:** 5-year survival probabilities for cancers of children with and without Turkish descent, German Childhood Cancer Registry 1980–2005 by sex and age at diagnosis.

	5 year survival probability % (number of deaths within 5 years of diagnosis)		
	Turkish descent	Non-Turkish	p-value, log-rank test
**All Malignancies**	76.9 (400)	77.6 (7823)	0.15
Boys	77.1 (233)	77.0 (4483)	0.45
Girls	76.6 (167)	78.3 (3340)	0.19

**Age at diagnosis**			
0–<1 years	77.5 (37)	77.1 (806)	0.90
1–<5 years	79.4 (138)	79.2 (2642)	0.69
5–<10 years	76.7 (114)	77.8 (2077)	0.21
10–<15 years	72.9 (111)	75.3 (2298)	0.18

Stratification by diagnosis group (see Additional file 2) showed generally very similar diagnosis-specific survival probabilities for children of Turkish and non-Turkish descent. We found a statistically significant disadvantage for children with Turkish descent only for the group of leukaemias, myeloproliferative and myelodysplastic diseases (76.2% versus 78.4%; p logrank = 0.028), caused by the difference in the largest subgroup of lymphoid leukaemias (p logrank = 0.013).

**Figure 1:**
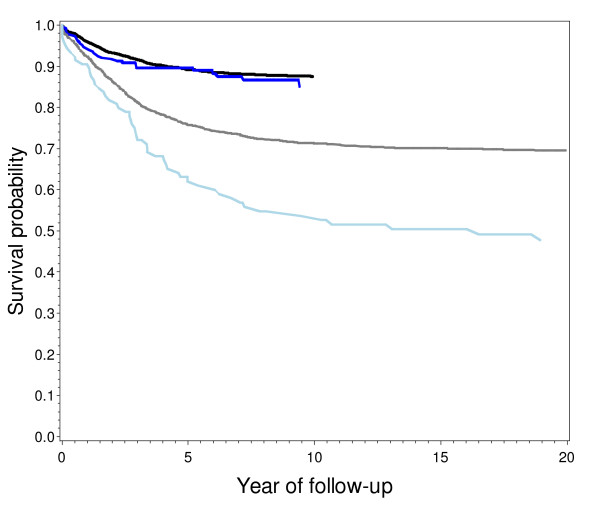
**Colour version: Kaplan-Meier estimate of survival curve of lymphoid leukaemia (ICCC3 Ia), cases with and without Turkish descent diagnosed 1980–87 and 1996–2005 followed up until 12/2006**. Black line: non-Turkish cases diagnosed 1996–2005 (507 events/4559 cases). Blue line: Turkish cases diagnosed 1996–2005 (32 events/265 cases). Grey line: non-Turkish cases diagnosed 1980–87 (719 events/2361 cases). Light blue line: Turkish cases diagnosed 1980–87 (52 events/105 cases).

Further stratification by time of diagnosis (see Additional file 3) revealed a significant overall survival disadvantage of children with Turkish migrant background (64.8% versus 67.8%, p = 0.016) for the period 1980–87, again driven by lymphoid leukaemia. The 5-year survival probability from lymphoid leukaemia diagnosed in the period 1980–87 for non-Turkish children was markedly higher (75.8%) as compared to the Turkish group (62.0%, p < 0.0001) with a rapid dissociation of the respective survival curves (see Figure 1). Further analyses (not shown) revealed no differences in morphological subgroups, age at diagnosis and completeness of follow-up for this particular group sufficiently large to explain this difference. Leukaemia cases of Turkish descent both with and without recorded recurrences had similar survival probabilities, but Turkish cases had more frequent recurrences. In the more recent periods, these differences have disappeared (see Figure 1). The 5-year survival probabilities for leukaemia for cases diagnosed 1996–2005 are remarkably similar between groups and now exceed 80% in both non-Turkish children and those of Turkish descent.

Based on small numbers, we found survival probability differences for some diagnosis subgroups and time periods of central nervous system tumours (e.g. astrocytomas: 1980–87 Turkish (3 events) 80%, Non-Turkish (109 events) 70%, p = 0.89; 1996–2005 Turkish (24 events) 70%, Non-Turkish (334 events) 78%, p = 0.067). Some further diagnoses show marked survival disadvantages (e.g other soft tissue sarcomas: 59% (9 events) vs 71% (173 events, p = 0.11) but also advantages (e.g. Rhabdomyosarcoma 78% (13 events) vs 69% (422 events, p = 0.50) in general or in some time periods for children with Turkish descent, but these are all non-significant due to the rarity of tumours and events.

## Discussion

Our data include information from more than 37,000 childhood cancer cases in Germany and show that Turkish and non-Turkish children in Germany nowadays do not differ with respect to survival from childhood cancer. This can be seen as a reflection of the high standard of care granted to children with cancer in Germany, a standard which allows equitable and accessible care to all patient groups irrespective of geographical descent as assessed in our study. The findings of highly comparable cancer survival probabilities are largely consistent across diagnostic, sex and age groups. Only during the early period of the GCCR, from 1980–1987, when registration was not yet quite complete, we did see significant survival disadvantages for Turkish children, largely confined to lymphoid leukaemia. The available data offer little clues why this difference occurred. So far we have no indication that incomplete reporting in the early 1980s was related to prognosis or migrant status, but a selection bias cannot be entirely excluded. Given the large number of statistical comparisons we performed, chance may be among the possible explanations. Problems that could be associated with e.g. a language barrier, such as parental compliance with therapy recommendations, appear unlikely, but may have played a role in certain specific diagnoses with therapeutic regimen that require long-term care after the initial therapy. There are, however, no recorded data to further assess this hypothesis. Socioeconomic status did not change very much for Turkish migrant groups over the time period investigated, neither did access to medical care, especially for children. Most of these potential explanatory factors would also have affected all diagnoses to the same degree. More important are the recent results showing very similar survival probabilities in the groups compared. The actual survival estimates for the most recent period may be overestimated for all cases due to incomplete follow-up.

Our study ranks amongst the largest investigations of migrant-specific cancer survival probability. It is based on the highly comprehensive nationwide German Childhood Cancer Registry. The mortality follow-up followed routinely established procedures and showed very similar results for both groups, with a surprisingly low loss to follow-up due to out-migration. Our approach to identify cases with Turkish ethnicity relies on the assumption that children with Turkish names indeed have a Turkish descent, and that there is no differential uptake of German names with regard to cancer prognosis. We cannot exclude a small bias through this mechanism. However, the number of mixed German-Turkish marriages has only increased in more recent years. A major proportion of these marriages occurs between partners of whom one has taken up German nationality, but is of Turkish descent. Thus, Turkish first or family names are likely to be retained in many mixed marriages. Children from mixed marriages with neither a Turkish first or last name are likely to be rare.

In our study we could not differentiate between different migrant generations. Potentially, such information could further elucidate the differences in survival probability seen in early phases of the GCCR, but such data are not likely to become available in registry – based studies. This latter comment is also true for information on the socioeconomic status of cases or their families, which is not routinely recorded in the registry.

While the overall number of deaths allows for relatively precise estimates, the stratifications by diagnosis and period we performed led to rather small numbers of cases in some strata. In addition, multiple comparisons should be noted, but since our study had an explorative character, no adjustment for multiple testing seemed warranted. Thus, some of the differences noted as statistically significant are possibly due to chance.

Due to the absence of routine data to assess cancer incidence and survival probability according to ethnic background, this is the first study in Germany on this topic, and one of the few investigations published in Europe so far. McKinney [8] assessed the cancer survival probability in a cohort of 1979 children with cancer in Yorkshire, using a name-based approach to identify children of South Asian origin. No survival probability differences were found between South Asian and other children, and indications that higher levels of deprivation were linked with lower survival probability were not statistically significant. In a large study of all childhood cancer cases between 1981 and 1996 in the UK, Stiller [9] found little evidence of ethnic differences in survival probabilities. The UK Children's Cancer Study Group register contains primary information on the ethnic group of patients, which allows comparisons across different ethnic groups. For acute lymphoid leukaemia, the authors reported slightly lower long-term survival probabilities for children of South Asian origin. Our own finding of survival probability differences in lymphoid leukaemia mirrors this observation in one of the largest migrant groups in Germany.

Several studies from the US report ethnic differences in cancer survival probability, most of them with a focus on leukaemia. In a series of cases from the St. Jude Children's Research Hospital spanning 30 years of incident diagnoses, Pui [10] found large survival disadvantages for black children in the early periods which disappeared in the 1980s and 1990s. A single centre study of 412 children with acute lymphoid leukaemia treated in Memphis also showed no differences in survival probability [11]. Conversely, Bhatia concluded from a review of available single-and multi centre studies that black children with acute lymphoid leukaemia continue to have lower survival probabilities than white children in the US [12]. For acute myeloid leukaemias a survival disadvantage of black children has recently been described based on experiences from two US Children's Oncology Group trials [13]. Interestingly, there was only a low proportion of black children with appropriate matched family donors for transplants.

Overall, the small body of studies available is relatively consistent with regard to cancer survival probability, showing generally few differences between cases with and without migrant background or by ethnic origin. Haematological cancers could be an exception, as, amongst others, the recent results from the US indicate. In our own study, differences in survival probabilities from lymphoid leukaemia were limited to children diagnosed between 1980 and 1987. Nevertheless, as leukaemias are the largest group of childhood cancers, continued surveillance of survival using updated markers of ethnic or geographical origin may be warranted.

## Conclusion

In summary, our study demonstrates increasing and very similar 5-year cancer survival probabilities among children with and without Turkish descent living in Germany. This finding is consistent with the small body of available information on overall cancer survival of ethnic minority children from other countries. We interpret this as showing the non-discriminative nature of current childhood cancer care in Germany and the provision of equitable access to modern therapeutic and follow-up regimes, irrespective of migrant status.

## Competing interests

The authors declare that they have no competing interests.

## Authors' contributions

HZ, OR and JS conceived the study. JS coordinated the study. CS performed the survival analysis and wrote the first draft of the manuscript. CS and PK provided the data. JS, OR and HZ participated in the design of the study and the data analysis. All authors helped to draft the manuscript and read and approved it in its final form.

## Pre-publication history

The pre-publication history for this paper can be accessed here:



## Supplementary Material

Additional file 1 **Diagnosis distribution and proportion of cases with Turkish descent by ICCC-3.** Listed are all ICCC-3 groups and all subgroups with at least 10 cases of Turkish descent. Click here for file

Additional file 2 **5-year survival probabilities for cancers of children with and without Turkish descent, German Childhood Cancer Registry 1980–2005, by ICCC-3 groups.** Follow up of cases until 31.12.2006 or until loss to follow up. Listed are all ICCC-3 groups and subgroups with at least 5 deaths among cases with Turkish descent. Click here for file

Additional file 3 **5-year survival probabilities for cancers of children with and without Turkish descent, German Childhood Cancer Registry 1980–2005 by time period of diagnosis.** Follow up of cases until 31.12.2006 or until loss to follow up. Listed are all ICCC-3 groups and all subgroups with at least 5 deaths among cases with Turkish descent per time period. Click here for file
