# Supplementary material for: Cancer survival among children of Turkish descent in Germany 1980–2005: a registry-based analysis
Source: BMC Cancer. 2008 Nov 28;8:355. doi: 10.1186/1471-2407-8-355 (PMC2628927; doi:10.1186/1471-2407-8-355)
Supplement: Additional file 1 — Diagnosis distribution and proportion of cases with Turkish descent by ICCC-3. Listed are all ICCC-3 groups and all subgroups with at least 10 cases of Turkish descent. [file 1471-2407-8-355-S1.doc]

Diagnosis distribution and proportion of cases with Turkish descent by ICCC-3

Listed are all ICCC-3 groups and all subgroups with at least 10 cases of Turkish descent

|  |  | Turkish descent  N | Non-Turkish  N | % of Turkish descent |
| --- | --- | --- | --- | --- |
| All Malignancies |  | 1774 | 35,485 | 4.8 |
| ICCC-3 groups | ICCC-3 subgroups |  |  |  |
| Leukaemias, myeloproliferative and myelodysplastic diseases |  | 658 | 12,624 | 5.0 |
|  | Lymphoid leukaemias | 506 | 10,177 | 4.7 |
|  | Acute myeloid leukaemias | 111 | 1827 | 5.7 |
|  | Chronic myeloproliferative diseases | 16 | 162 | 9.0 |
|  | Myelodysplastic syndrome and other myeloproliferative diseases | 22 | 345 | 6.0 |
| Lymphomas and reticuloendothelial neoplasms |  | 270 | 4331 | 5.9 |
|  | Hodgkin lymphomas | 119 | 1778 | 6.3 |
|  | Non-Hodgkin lymphomas | 120 | 2068 | 5.5 |
|  | Burkitt lymphoma | 24 | 369 | 6.1 |
| CNS and miscellaneous intracranial and intraspinal neoplasms |  | 318 | 6679 | 4.5 |
|  | Ependymomas and choroid plexus tumour | 36 | 714 | 4.8 |
|  | Astrocytomas | 135 | 2737 | 4.7 |
|  | Intracranial and intraspinal embryonal tumours | 92 | 1770 | 4.9 |
|  | Other gliomas | 20 | 439 | 4.4 |
|  | Other specified intracranial and intraspinal neoplasms | 29 | 758 | 3.7 |
| Neuroblastoma and other peripheral nervous cell tumours |  | 143 | 2890 | 4.7 |
|  | Neuroblastoma and ganglioneuroblastoma | 143 | 2874 | 4.7 |
| Retinoblastoma |  | 28 | 804 | 3.4 |
| Renal tumours |  | 95 | 2199 | 4.1 |
|  | Nephroblastoma and other non-epithelial renal tumours | 92 | 2164 | 4.1 |
| Hepatic tumours |  | 17 | 365 | 4.5 |
|  | Hepatoblastoma | 13 | 290 | 4.3 |
| Malignant bone tumours |  | 79 | 1748 | 4.3 |
|  | Osteosarcomas | 37 | 933 | 3.8 |
|  | Ewing tumour and related sarcomas of bone | 39 | 767 | 4.8 |
| Soft tissue and other extraosseous sarcomas |  | 102 | 2294 | 4.3 |
|  | Rhabdomyosarcomas | 62 | 1372 | 4.3 |
|  | Other specified soft tissue sarcomas | 23 | 606 | 3.7 |
| Germ cell tumours, trophoblastic tumours and neoplasms of gonads |  | 48 | 1140 | 4.0 |
|  | Malignant extracranial and extragonadal germ cell tumours | 15 | 339 | 4.2 |
|  | Malignant gonadal germ cell tumours | 26 | 470 | 5.2 |
| Other malignant epithelial neoplasms and malignant melanomas |  | 16 | 376 | 4.1 |
| Others and unspecified malignant neoplasms |  | 0 | 34 | 0.0 |
| Not malignant |  | 0 | 1 | 0.0 |
