# Supplementary material for: Cancer survival among children of Turkish descent in Germany 1980–2005: a registry-based analysis
Source: BMC Cancer. 2008 Nov 28;8:355. doi: 10.1186/1471-2407-8-355 (PMC2628927; doi:10.1186/1471-2407-8-355)
Supplement: Additional file 2 — 5-year survival probabilities for cancers of children with and without Turkish descent, German Childhood Cancer Registry 1980–2005, by ICCC-3 groups. Follow up of cases until 31.12.2006 or until loss to follow up. Listed are all ICCC-3 groups and subgroups with at least 5 deaths among cases with Turkish descent. [file 1471-2407-8-355-S2.doc]

5-year survival probabilities for cancers of children with and without Turkish descent, German Childhood Cancer Registry 1980-2005, by ICCC-3 groups. Follow up of cases until 31.12.2006 or until loss to follow up. Listed are all ICCC-3 groups and subgroups with at least 5 deaths among cases with Turkish descent

|  | | 5 year survival probability % (number of deaths within 5 years of diagnosis) | |  |
| --- | --- | --- | --- | --- |
|  | | Turkish descent | Non-Turkish | p-value,  log-rank test |
| **ICCC-3 groups** | **ICCC-3 subgroups** |  |  |  |
| Leukaemias, myeloproliferative and myelodysplastic diseases |  | 76.2 (153) | 78.4 (2677) | 0.028 |
|  | Lymphoid leukaemias | 82.3 (87) | 84.3 (1558) | 0.013 |
|  | Acute myeloid leukaemias | 53.6 (51) | 52.5 (859) | 0.76 |
|  | Chronic myeloproliferative diseases | 55.6 (7) | 54.7 (72) | 0.91 |
|  | Myelodysplastic syndrome and other myeloproliferative diseases | 71.1 (6) | 60.7 (133) | 0.38 |
| Lymphomas and reticuloendothelial neoplasms |  | 88.6 (30) | 89.3 (460) | 0.39 |
|  | Hodgkin lymphomas | 92.3 (9) | 96.2 (65) | 0.070 |
|  | Non-Hodgkin lymphomas | 87.1 (15) | 84.6 (316) | 0.94 |
| CNS and miscellaneous intracranial and intraspinal neoplasms |  | 65.4 (108) | 69.8 (1976) | 0.11 |
|  | Ependymomas and choroid plexus tumour | 76.1 (8) | 68.5 (216) | 0.39 |
|  | Astrocytomas | 71.8 (38) | 76.0 (648) | 0.22 |
|  | Intracranial and intraspinal embryonal tumours | 53.4 (42) | 60.2 (693) | 0.29 |
|  | Other gliomas | 37.5 (12) | 46.4 (231) | 0.52 |
| Neuroblastoma and other peripheral nervous cell tumours |  | 70.8 (41) | 69.6 (865) | 0.83 |
|  | Neuroblastoma and ganglioneuroblastoma | 70.8 (41) | 69.5 (862) | 0.82 |
| Renal tumours |  | 90.5 (9) | 87.9 (263) | 0.59 |
|  | Nephroblastoma and other non-epithelial renal tumours | 90.2 (9) | 87.9 (259) | 0.67 |
| Hepatic tumours |  | 64.7 (6) | 64.0 (130) | 0.74 |
|  | Hepatoblastoma | 69.2 (4) | 71.4 (82) | 0.44 |
| Malignant bone tumours |  | 72.2 (21) | 68.6 (535) | 0.28 |
|  | Osteosarcomas | 80.1 (7) | 79.7 (265) | 0.12 |
|  | Ewing tumour and related sarcomas of bone | 62.8 (14) | 66.4 (252) | 0.82 |
| Soft tissue and other extraosseous sarcomas |  | 73.3 (26) | 69.2 (692) | 0.90 |
|  | Rhabdomyosarcomas | 78.2 (13) | 68.7 (422) | 0.50 |
|  | Other specified soft tissue sarcomas | 58.8 (9) | 70.9 (173) | 0.11 |
| Other malignant epithelial neoplasms and malignant melanomas |  | 64.5 (5) | 77.8 (81) | 0.20 |

ICCC International Classification of Childhood Cancer, CNS central nervous system
