# Supplementary material for: Cancer survival among children of Turkish descent in Germany 1980–2005: a registry-based analysis
Source: BMC Cancer. 2008 Nov 28;8:355. doi: 10.1186/1471-2407-8-355 (PMC2628927; doi:10.1186/1471-2407-8-355)
Supplement: Additional file 3 — 5-year survival probabilities for cancers of children with and without Turkish descent, German Childhood Cancer Registry 1980–2005 by time period of diagnosis. Follow up of cases until 31.12.2006 or until loss to follow up. Listed are all ICCC-3 groups and all subgroups with at least 5 deaths among cases with Turkish descent per time period. [file 1471-2407-8-355-S3.doc]

5-year survival probabilities for cancers of children with and without Turkish descent, German Childhood Cancer Registry 1980-2005 by time period of diagnosis

Follow up of cases until 31.12.2006 or until loss to follow up. Listed are all ICCC-3 groups and all subgroups with at least 5 deaths among cases with Turkish descent per time period

|  |  | 5 year survival probability % (number of deaths within 5 years of diagnosis) | |  |
| --- | --- | --- | --- | --- |
|  |  | Turkish descent | Non-Turkish | p-value,  log-rank test |
| **All malignancies** |  |  |  |  |
| 1980-87 |  | 64.8 (128) | 67.8 (2426) | 0.016 |
| 1988-95 |  | 74.7 (120) | 76.2 (2695) | 0.40 |
| 1996-2005 |  | 83.1 (152) | 83.1 (2702) | 0.78 |
| ICCC-3 groups | ICCC-3 subgroups |  |  |  |
| Leukaemias, myeloproliferative and myelodysplastic diseases |  |  |  |  |
| 1980-87 |  | 54.0 (63) | 68.8 (908) | <0.0001 |
| 1988-95 |  | 75.4 (43) | 77.4 (896) | 0.46 |
| 1996-2005 |  | 86.2 (47) | 84.2 (873) | 0.75 |
|  | Lymphoid leukaemias |  |  |  |
|  | 1980-87 | 62.0 (39) | 75.8 (571) | <0.0001 |
|  | 1988-95 | 84.6 (21) | 84.1 (519) | 0.82 |
|  | 1996-2005 | 89.7 (27) | 89.2 (468) | 0.55 |
|  | Acute myeloid leukaemias |  |  |  |
|  | 1980-87 | 32.1 (19) | 38.5 (272) | 0.39 |
|  | 1988-95 | 43.3 (17) | 48.8 (295) | 0.78 |
|  | 1996-2005 | 71.6 (15) | 63.0 (292) | 0.24 |
| Lymphomas and reticuloendothelial neoplasms |  |  |  |  |
| 1980-87 |  | 82.2 (11) | 80.8 (180) | 0.46 |
| 1988-95 |  | 89.2 (7) | 90.2 (140) | 0.80 |
| 1996-2005 |  | 91.3 (12) | 92.7 (140) | 0.68 |
|  | Non-Hodgkin lymphomas |  |  |  |
|  | 1980-87 | 77.0 (5) | 72.7 (119) | 0.60 |
|  | 1988-95 | 84.6 (4) | 87.3 (91) | 0.41 |
|  | 1996-2005 | 91.5 (6) | 88.2 (106) | 0.36 |
| CNS and miscellaneous intracranial and intraspinal neoplasms |  |  |  |  |
| 1980-87 |  | 65.5 (18) | 56.4 (486) | 0.50 |
| 1988-95 |  | 58.6 (37) | 67.0 (676) | 0.21 |
| 1996-2005 |  | 69.1 (53) | 76.0 (814) | 0.045 |
|  | Astrocytomas |  |  |  |
|  | 1980-87 | 80.0 (3) | 69.7 (109) | 0.89 |
|  | 1988-95 | 73.6 (11) | 74.4 (201) | 0.96 |
|  | 1996-2005 | 69.2 (24) | 78.2 (338) | 0.067 |
|  | Intracranial and intraspinal embryonal tumours |  |  |  |
|  | 1980-87 | 76.5 (4) | 45.5 (182) | 0.040 |
|  | 1988-95 | 25.3 (22) | 54.2 (270) | 0.0031 |
|  | 1996-2005 | 63.6 (16) | 70.7 (241) | 0.34 |
| Neuroblastoma and other peripheral nervous cell tumours |  |  |  |  |
| 1980-87 |  | 50.0 (11) | 53.4 (281) | 0.66 |
| 1988-95 |  | 66.8 (15) | 65.1 (320) | 0.83 |
| 1996-2005 |  | 79.3 (15) | 79.9 (264) | 0.83 |
|  | Neuroblastoma and ganglio neuroblastoma |  |  |  |
|  | 1980-87 | 50.0(11) | 53.1 (281) | 0.67 |
|  | 1988-95 | 66.8 (15) | 65.0 (319) | 0.81 |
|  | 1996-2005 | 79.3 (15) | 79.9 (262) | 0.83 |
| Malignant bone tumours |  |  |  |  |
| 1980-87 |  | 65.6 (9) | 61.9 (178) | 0.41 |
| 1988-95 |  | 70.0 (6) | 65.4 (181) | 0.63 |
| 1996-2005 |  | 78.7 (6) | 75.4 (176) | 0.44 |
| Soft tissue and other extraosseous sarcomas |  |  |  |  |
| 1980-87 |  | 63.6 (8) | 60.9 (196) | 0.61 |
| 1988-95 |  | 68.8 (7) | 67.4 (247) | 0.49 |
| 1996-2005 |  | 79.9 (11) | 74.9 (249) | 0.42 |
|  | Rhabdomyosarcomas |  |  |  |
|  | 1980-87 | 72.7 (3) | 57.2 (135) | 0.83 |
|  | 1988-95 | 71.4 (4) | 69.6 (138) | 0.61 |
|  | 1996-2005 | 83.0 (6) | 74.0 (149) | 0.25 |

Note: The follow-up in the last diagnosis period is as yet incomplete; survival probabilities may be overestimated
